# Obstetrics

**Published:** 1875-11

**Authors:** 


### Summary of Progress in the Medical Sciences.

### I. Obstetrics.

1. *Chloral as an Anæsthetic in Natural Labor.* By Dr. H. Chauppe. (*Annales de Gynécologie*, Mai, 1875.)

The points considered by the writer are:

1. *Obsterical anæsthesia* CAN *be produced by chloral;*
2. *It is without danger to mother and child;* and
3. *In what cases, at what period of labor, in what doses, form, and by what channel it ought to be administered.*

I. The writer has collected thirty-seven cases of the use of chloral thus: "In thirty cases anæsthesia was *complete;* in five cases it was *almost* complete; and in two cases it was *not* produced." Twelve of these cases are given at length, a few of which are here given at random.

"Case VI. L'hôpital de la Charité; service of M. Bourdon, Dec. 17, 1873; patient aged 23; labor commenced at midnight; at 1 A. M. her pains were very severe. The cervix was dilated to the extent of a two-franc piece (quarter of a dollar); vomiting. Two grammes (about thirty grains) of chloral hydrate were administered, but this potion provoked vomiting anew.

"9.10 A. M. Two grammes (thirty grs.) of chloral were given per rectum.

"9.20 A.M. She commenced to sleep, although she cried out at the time of the contractions.

"9.40 A. M. A new injection of two grammes of chloral given. From this time the patient was calm, the contractions continued to be produced without any pain till 11.30 A. M., at which time the delivery was completed. During the labor the patient awakened at the time of the contractions, but she did not suffer.

"She left the hospital in perfect health at the end of eight days."

"Case VIII. Feb. 28. Mrs. V., æt. 22, primipara. Had been in labor since evening of the 26th inst. The cervix, dilated to size of a two franc piece, was thin and rigid. Since the evening of the 27th inst. she had been in the same condition. She had had administered to her a tepid bath, opiates and emetics, without any result.

"8.05 A. M. Three grammes (45 grs.) of chloral hydrate given, the pulse beating 100 per minute.

"9 A. M. Sleeping. Pulse, 90.

"9.10 A. M. Patient continues sleeping. Pulse, 88.

"9.40 A. M. Patient is aroused for a moment; she had a few slight pains and almost immediately sleep again supervened.

"10 A. M. Cervix in same state; sleeping lightly.

"10.13 A. M. Patient agitated a little during a contraction. One gramme of chloral given. The patient had now taken four grammes in an hour and a half.

"After the last dose she fell asleep again profoundly. The sleep was prolonged till 3 o'clock, at which time dilatation was complete, and the expulsive pains commenced and delivery was rapidly terminated without pain. Recovery complete."

"Case X. Rosalie B., æt. 25 years, primipara, in perfect health, arrived at full term without a single accident. The day previous, 3 P. M., she began to experience the first pains, which lasted throughout the night, causing her to cry out. At 8.30 A. M. on the day following, the cervix was completely dilated, the head, fixed, was engaged in the superior strait in the first position.

"The contractions were irregular and weak. The labor had not progressed since 5 A. M. Four grammes of chloral were given; the first two at once, and the last two separately, a quarter of an hour apart; after the second dose the patient slept and had only one contraction, which did not awaken her, between that and the third dose. After the fourth dose, powerful contractions came on very regularly every five minutes, without arousing the patient, and at the end of thirty-five minutes delivery was completed without her remarking it. At the moment of delivery she pretended not to have suffered and then fell asleep and slept five hours, waking perfectly refreshed. The sequences were as usual, and she left the hospital on the tenth day after confinement."

"Case XII. Céleste K., æt. 24 years, primipara, entered the hospital Dec. 4, 1873; is at full term, which she reached without any accident. The pains began two days previous, and waters had escaped twelve hours previously.

"This woman, very nervous, was continually crying out and tossing about on the bed; she said she suffered much; contractions irregular; cervix completely dilated, breech presentation; first presentation at the superior strait.

"8.40 A. M. Two grammes of chloral given at once.

"8.55 A. M. No effect; two more grammes given.

"9.05 A. M. No effect; perhaps there is a slight tendency to sleep and the cries a little less violent, but the contractions are not at all modified. One gramme more given.

"9.15 A. M. A little tendency to sleep. One gramme more given.

"9.25 A.M. No complete sleep yet; the seventh gramme given.

"9.45 A. M. After the last dose the patient fell into a profound, very calm sleep; the pulse which had been 120 beats per minute, fell to 80; respiration calm, but the uterus did not contract.

"9.50 A. M. Contractions violent, without arousing the patient. From this moment the contractions became regular and powerful; at first every six minutes, then every five minutes, and the delivery was consummated without intervention at 11.10 A. M. The patient was not conscious one moment. She slept till 2 P. M., at which time she awakened and said she remembered nothing, and had not suffered any. Recovery was rapid."

An attentive reading of the above given cases, will convince any one that this drug administered as shown, places in the obstetrician's hands a help scarcely second to chloroform, and one free from the objections of the latter, which every one knows "prolongs the labor" by rendering the pains less effective. The author claims for this drug, that it not only does *not* protract labor as chloroform does, but it positively *increases* pains, steadies irregular, ineffectual contractions, and at once converts them into forcible, expulsive uterine efforts. To carry conviction still more forcibly to his readers, the writer details two experiments, on bitches, performed by M. Pellissier. The dogs were fastened to the operating table, the abdomen opened and at first the contact of air sufficed to produce contractions of the unstriped fibres of the intestines and uterus. Pinching these organs produced violent muscular contractions. Into one animal chloral was injected till profound sleep was produced. "Anæsthesia was complete; the nose and tongue were pinched without producing a single movement." Slightly pinching the uterus produced contraction promptly. Into the other bitch laudanum was injected subcutaneously; soon "very profound sleep, with violent dyspnœa, was produced." Upon pinching, the intestine and uterus contracted, "but they had to be observed with the *utmost attention* to see these contractions, since they were *scarcely perceptible.*" After these experiments, both animals recovered; this fact is mentioned purposely, to assure any incredulous reader that these experiments were not prosecuted on moribund subjects.

Obstetrical anæsthesia involves two points, viz., *abolition of suffering* and the *maintaining the integrity of the uterine contraction.* The former any observer will concede. As to the second point, the writer triumphantly remarks: "Case XII, for example, is one of the best examples we can take. What does this case show us? We have a very nervous, fatigued woman; the presentation is bad, the labor of long duration; the pains succeed each other rapidly and are ineffectual. Chloral is given. Immediately the uterus ceases to contract, and, one would believe, that, at the same time that anæsthesia is induced, the medicament had arrested the uterine contractions. But the illusion is not of long duration; to the painful, powerless contractions which the patient suffers, regular energetic contractions succeed, which are produced without pain, and, in a short time, accomplish the expulsion of the child. If we analyze the twelve cases, *in extenso*, we see, with few exceptions, the same thing produced in all the cases."

"We now believe it to have been fully established, that chloral hydrate, which is capable of producing in the highest degree, cutaneous anæsthesia, can as effectually produce an annihilation of the pains of confinement; that this suppression of pain is not due to the diminution of uterine contractions, either in their intensity or frequency ; that the latter (the pains) upon the contrary conserve their power ; that they seem for a few moments to be retarded, and that what they lose in frequency they gain in force. This important verification explains why some authors have believed that chloral acts directly upon the uterine fibres as an excitant ; it explains why M. Lambert, without enunciating a similar opinion, could terminate his essay by saying, 'that by the use of chloral, labor is not only *not* retarded, but it is often accelerated.' "

II. *Effect on Mother and Child.*

It is well established that chloral does not diminish the contractions nor retard labor. M. Pellissier has always examined with great care the children born of chloralized mothers, and "has never seen the former show in the least degree the effect of the hypnotic and anæsthetic agent. He has never seen the milk of the mother, given to the infant almost immediately after birth, produce the least hypnotic effect." He affirms, after using it many times, that "chloral administered in a prudent manner and in proper doses, is an agent of absolute innocuity."

The author has been impressed with the "rapidity of recovery" of the women, chloralized. He admits that his collection may have been "a happy series" of cases, and this rapidity exceptional, rather than the rule. If it be the latter, he suggests two solutions, inclining to the latter. 1st. It may be owing to the escape of the mother from the great fatigue of labor. 2nd. It may be owing "to the destroying or neutralizing of the septic products absorbed by the placental wound." Since chloral solutions are applied to ulcers and wounds on account of the antiseptic, antiputridic properties of the drug which are now well established, the author suggests that, during the first few hours after expulsion of the fœtus, the chloral in the blood acts as a disinfectant. He advances this suggestion "with the most extreme reserve," and promises to make this point a special study.

III. *In what cases, when, in what dose, and by what channel ought it to be administered.*

"Chloral ought especially to be given in too prolonged confinements and in primiparas, for then the pains have an intensity that will be found only exceptionally in ulterior confinements." "It often occurs that, the pains being very intense, the patients, when they are nervous, are exhausted by their inutile efforts, and that at the moment when they especially need all of their forces for the last expulsive pains, they are fatigued and the womb ceases to contract. In these cases, chloral, which procures repose and rescues the women from exhaustion, would be expressly indicated."

"It will be still more appropriate in nervous women, who toss about and complain beyond measure. If anæsthesia can be induced in such patients, the moment of delivery will be remarkably hastened."

"In hysterical females, who are so often attacked at the moment of parturition, chloral can be used with success."

After pains can be controlled with chloral.

At what time of labor ought chloral to be used? As a general proposition, the author would recommend that it be given "when dilatation is complete, and when the expulsive pains have commenced." . . . "It is only in very rare cases and when the woman suffers much that we would be authorized in giving it during the period of dilatation."

The dose will vary, "according to the moment of giving it, the susceptibility of the patient and the effect desired." The dose varies from 60 to 80 grains, given in 30 or 40 grs. at an interval of 30 minutes, or, in 15-grain doses every 15 minutes, till the effect is produced.

When the stomach will not retain it, the rectum should be resorted to. The hypodermic and intravenous uses of chloral the author would not recommend.

